# Comparative transcriptomic analysis of tumor- infiltrating canine natural killer cells and candidate biomarkers from first-in-dog NK immunotherapy trials

**DOI:** 10.3389/fimmu.2025.1646849

**Published:** 2025-10-24

**Authors:** Aryana M. Razmara, Marshall Lammers, Sean J. Judge, Cyrus J. Sholevar, William J. Murphy, Cameron E. Gaskill, William T. N. Culp, Alicia A. Gingrich, Zachary S. Morris, Robert B. Rebhun, C. Titus Brown, David M. Vail, Michael S. Kent, Robert J. Canter

**Affiliations:** ^1^ Division of Surgical Oncology, Department of Surgery, University of California Davis School of Medicine, Sacramento, CA, United States; ^2^ Department of Dermatology, University of California Davis School of Medicine, Sacramento, CA, United States; ^3^ Department of Surgical and Radiological Sciences, University of California Davis School of Veterinary Medicine, Davis, CA, United States; ^4^ Department of Human Oncology, University of Wisconsin School of Medicine and Public Health, Madison, WI, United States; ^5^ Department of Population Health and Reproduction, University of California Davis School of Veterinary Medicine, Davis, CA, United States; ^6^ Department of Medical Sciences, University of Wisconsin School of Veterinary Medicine, Madison, WI, United States

**Keywords:** natural killer (NK) cells, cancer immunotherapy, single-cell RNA sequencing (scRNAseq), canine sarcoma infiltrating NK signature, tumor infiltrating NK cells, canine and human sarcomas, NK-targeting immunotherapy regimens, NK activation genes

## Abstract

**Introduction:**

Natural killer (NK) cells have great potential to extend the promise of cancer immunotherapy, but additional research is needed to improve their efficacy in solid cancers. Dogs develop spontaneous cancers with striking similarities to humans and can serve as a crucial link to bridge murine studies and human clinical trials to improve treatment outcomes across species and identify potential biomarkers of response.

**Methods:**

Using single-cell RNA sequencing (scRNAseq), we integrated blood, tissue, and tumor samples from dog and human donors to compare NK cell gene expression and develop a canine sarcoma infiltrating NK signature. Canine tissue and tumor NK cell signatures were then used to contextualize NK cell changes in first-in-dog immunotherapy clinical trials.

**Results:**

Tumor infiltrating NK cells from both canine and human sarcomas exhibited enhanced migration with a simultaneously exhausted signature that most closely correlated transcriptionally with NK cells isolated from the liver. We also analyzed peripheral blood NK cells from dogs on first-in-dog clinical trials undergoing three distinct NK-targeting immunotherapy regimens, observing that dogs with favorable responses demonstrated increased NK proportions posttreatment. Genes upregulated in NK cells in the peripheral blood of good responders included genes associated with activated NK cells and revealed post-treatment gene expression changes in the blood as a predictor of response.

**Discussion:**

Overall, NK effector functions are well adapted to their tissue of residence but dysregulated in sarcoma infiltrating NK cells despite enhanced migration. We describe NK cell trends across canine clinical trials as a platform through which we can elucidate mechanisms of response and determine novel immunotherapy strategies to improve cancer outcomes in both humans and dogs.

## Introduction

NK cells are attractive candidates for cancer immunotherapy given their ability to spontaneously target diverse cancer cells that have evaded other immune recognition strategies and their minimal association with toxicity. NK cell therapies, including recent advances in CAR NK cells, have significantly improved outcomes for patients with leukemias and lymphomas (1, 2). However, the complex tumor microenvironment (TME) of solid tumors introduces additional challenges in cancer immunotherapy, including the presence of exhausted NK cells in the TME (3). Thus, further work is needed to realize more impactful success of NK cells in the treatment of solid cancers.

Importantly, dogs are an outbred species that develop cancers spontaneously in the context of an intact immune system, supporting their strength as a valuable model of human malignancy. This is especially true given the genotypic and phenotypic resemblance of spontaneous canine cancers to human cancers including immunoediting and immune surveillance with data showing homology between dog and human lymphoma, osteosarcoma, mammary tumors, melanoma, and high-grade gliomas (4–12). Consequently, the canine comparative oncology population is actively being leveraged to inform human cancer treatments, including novel immunotherapy combination trials for osteosarcoma and melanoma (6, 13, 14).

Our group previously published a thorough single-cell atlas of canine NK cells across blood and tissues including a comparative analysis with human NK cell abundances and cell-to-cell interactions (15). We identified tissue-specific NK signatures and comprehensive NK subsets using single-cell RNA sequencing (scRNAseq), allowing for extensive gene expression analysis to circumvent current limitations in canine reagents and lack of a markers unique to canine NK cells. Similar to humans, those data highlight the heterogeneity of canine NK cells across organs and tissue compartments and can inform analyses of tumor infiltrating NK cells, including in samples obtained from dogs on clinical trials with linkage to their outcomes. With our completion of multiple first-in-dog clinical trials using adoptive NK cell therapy, we have unique access to samples and outcomes of dogs undergoing NK targeting immunotherapy. While the responses to treatments and related bulk cell analyses of these trials have already been described (16), an understanding of how NK cell populations uniquely change in response to therapy has not been completed. The integration of cell-specific changes across trials and analysis of gene expression in the context of response outcomes has important implications for determining good gene candidates for optimizing therapy and identifying biomarkers of response to generate new directions for future therapies.

To bridge the newly uncovered canine NK cell signatures across healthy tissues to our clinical trial samples, we sought to analyze tumor-infiltrating NK cells from canine and human soft tissue sarcomas. Consequently, we determined an NK signature of soft tissue sarcoma (STS) in dogs with comparison to healthy tissue with direct comparison of human tissues and tumor for comparative context. Taking advantage of access to patient samples from three first-in-dog canine immunotherapy trials, we also performed single cell analysis of circulating NK cells from dogs in these trials. We noted both tissue and tumor-unique profiles conserved across species, and we observed dysregulated gene signatures in sarcoma-infiltrating NK cells with enhanced migration signatures as well as features of both simultaneous activation and inhibition. Finally, we demonstrate that dogs with favorable response to NK targeted immunotherapies have significant upregulation of NK activation genes in peripheral blood NK cells with implications for outcomes in human sarcoma patients. Collectively, our findings establish a landscape for evaluating tumor infiltrating NK cell genotypes compared to those of tissue resident NK cells and begin to unravel the mechanisms underpinning NK plasticity and heterogeneity in healthy and cancer-bearing dogs with relevance to humans.

## Methods

### Sample acquisition and processing

Tumor tissue was obtained with owner consent from residual tissue following resection of a soft tissue sarcoma, identified as a spindle cell sarcoma by histopathology from a 7-year-old male castrated boxer at the UC Davis Veterinary Medical Teaching Hospital. Sarcoma tissue was mechanically dissociated followed by incubation with RBC lysis buffer for five minutes at 4°C. PBMCs were isolated from whole blood from dogs undergoing immunotherapy trials using density gradient centrifugation (Lymphocyte Separation Medium, Corning Life Sciences), followed by red blood cell lysis. IACUC approval and signed informed owner consent were obtained for all dogs enrolled in clinical trials (IACUC Protocols #21461, #21620) (16). Processing was performed as described previously (16–18). The single-cell suspensions of PBMCs and tumor tissues were then ready for future submission for scRNA sequencing.

### Fluorescence-activated cell sorting

After tissue processing, canine tumor cells were washed with PBS and staining buffer, incubated with Fc receptor blocking solution (Canine Fc Receptor Binding Inhibitor, Invitrogen #14-9162-42), and stained with rat anti-dog monoclonal antibody CD45-EF450 (clone YKIX716.13, Invitrogen #48-5450-42). Live/dead discrimination was performed using Fixable Viability Dye 780. Cell sorting for live CD45+ cells was performed using the Becton Dickinson “Aria II” Cell Sorter (Becton Dickinson, San Jose, California, USA).

### Single-cell RNA sequencing and analysis

Library preparation and sequencing using the 10X Chromium Next GEM Single-Cell 3’ V.3.1 Gene Expression protocol performed by the UC Davis Genome Center as previously described (16). A canine (CanFam3.1) index was created using the *cellranger mkgtf* and *cellranger mkref* pipelines. Raw fastq files were aligned to the canine reference genome and the feature-barcode matrix was created using CellRanger v.7.1.0 (10x Genomics) before being uploaded in Rstudio and analyzed using Seurat. Seurat objects were created with a minimum cell threshold of 3 and minimum features of 200. Only cells with ≤15% of mitochondrial counts and unique feature counts ≥200 and ≤5,000-6,000 were filtered for analysis. Canine tumor cells also had filtering for PTPRC>0. Data then underwent standard Seurat processing workflow which included normalization, identification of highly variable features (2,000), and scaling. Cells were then clustered through a standard workflow that included linear dimensional reduction, determination of the k-nearest neighbor (KNN) using the top PCs based on the generation and interpretation of an elbow plot, and then implementation using a resolution of 0.5 after testing of multiple PCs and resolutions. Doublets were then identified and removed using DoubletFinder before the cell clustering workflow was repeated with doublets and unwanted cells removed. Cells were annotated manually based on markers and gene lists from relevant literature as well as by using the AddModuleScore function. NK cells in particular were identified as expressing NK genes such as KLRK1 and GZMA while simultaneously lacking expression of CD3E.

For merged analyses, samples were integrated using Harmony (19). Layers were joined and cells clustered using 50 PCs and resolution of 2. For subset datasets, cells identified as NK cells were subset followed by normalization, identification of variable features, scaling, PCA, clustering and generation of UMAP. For comparison between human and canine NK cells, human gene symbols were converted to canine using the convert_orthologs() function prior to integration. Differential gene expression testing was performed using the FindAllMarkers function in the Seurat R package to identify differences between two identified groups using the Wilcoxon Rank Sum test. Genes were only considered significant if the adjusted p-value, using Bonferroni correction, was p<0.05.

The tumor NK signature was developed by determining significantly different genes between NK cells within the tumor compared to NK cells in all remaining tissues and blood. The list of DEGs was then filtered to only include genes that had an adjusted p-value <0.05, average log fold change >1.0, and had expression in at least 20% of NK cells. Representative genes were selected and categorized by associations obtained from public databases (EnrichR, Uniprot, and NCBI).

Correlations were determined by extracting the scaled gene expression from merged NK cell datasets, then the Cor function was used to find pairwise correlation coefficients and create a correlation matrix. The correlation matrix was then visualized using CorrPlot with hierarchical clustering to order the variables.

### Publicly available data

Sample scRNAseq data from canine and human blood and tissue was obtained from publicly available database and analyzed as previously described (15). We also downloaded data analyzing single cell composition of the human STS tumor microenvironment previously published by Subramanian et al. using GEO accession code GSE212527 (20). Barcodes, features, and matrix files for sample ID SRC141, referencing a single UPS tumor, were available after data processing and read alignment by the authors using CellRanger v6.0.0 (10x Genomics) and human genome assembly GRCh38. These files were uploaded to R and used to create a Seurat object subject to the same workflow used for canine tumor. Additionally, we retrieved clinical and RNA expression data from the Cancer Genome Atlas (TCGA)- SARC or the TCGA- Melanoma dataset using the UCSC Xena platform (21). Specifically for the sarcoma dataset, all samples with the histological type of Undifferentiated Pleomorphic Sarcoma (UPS), Giant cell MFH/Undifferentiated pleomorphic sarcoma with giant cells, or Pleomorphic MFH/Undifferentiated pleomorphic sarcoma were included. Duplicate patients were removed to include only primary tumor samples. The median was used to determine two groups of high and low gene expression. Kaplan-Meier curves were based on overall survival and compared using the log-rank test.

## Results

### Canine sarcoma infiltrating NK cells are heterogeneous with unique activation and exhaustion features

**Figure 1:**
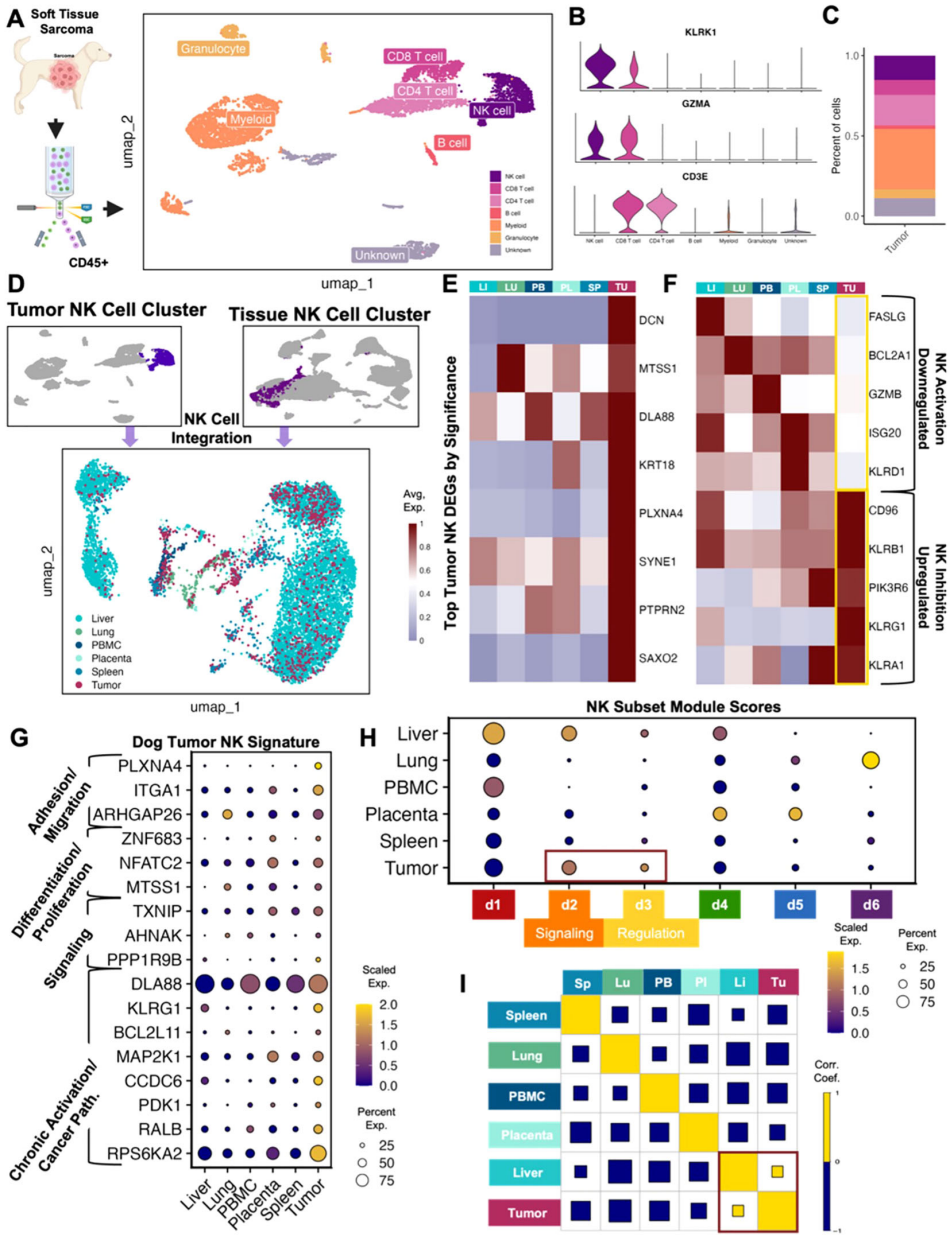
Canine tumor sarcoma infiltrating NK cells are heterogeneous with unique activation and exhaustion features. **(A)** Schema depicting workflow for FACS sorting and sequencing of CD45+ canine spindle cell sarcoma cells with the cell types identified. A total of 6,514 CD45+ immune cells were available for analysis. **(B)** Violin plot of key genes identifying NK cells and distinguishing them from T lymphocytes. **(C)** Bar plot showing the percent of each cell type in the tumor. NK cells represented 15.2% of all CD45+ tumor cells isolated. **(D)** NK cells subset from the canine tumor sample and NK cells subset from the integrated liver lung, PBMC, placenta, and spleen dataset were integrated and visualized by UMAP color coded by tissue. **(E)** Heatmap showing the average scaled expression of eight of the top differentially expressed genes in tumor NK cells that distinguish them from NK cells in all other tissues. **(F)** Heatmap of conventional NK activating and inhibitory genes and their average expression in tissues. Tumor NK expression is outlined. **(G)** Dotplot showing expression of representative genes significantly upregulated in tumor NK cells compared to NK cells in all other tissues. Genes included were present in >20% of cells, with average log2FC>1 and adjusted p-value<0.05. Gene category labels were determined by gene-set library and gene ontology annotations associated with each gene. **(H)** Dotplot visualization of the scaled module score of tissue-specific NK cells scored with the top genes that defined each canine NK subcluster. Subclusters with the highest expression in tumor NK cells were outlined and labeled. **(I)** Correlations across all tissue compartments. Yellow and blue squares represent positive and negative correlations respectively. Square size represents the absolute value of the correlation coefficient.

Interpreting the unique characteristics of immune cells of the TME is critical for understanding potential targets and improving cancer immunotherapy. To place our previously reported tissue-specific canine NK signatures in the context of tumor-resident immune cells, we analyzed immune cells from a canine soft tissue sarcoma. The dissociated tumor sample was sorted for CD45+ immune cells and analyzed by scRNAseq to identify cell types and their proportions (**Figures 1A–C**). A total of 6,514 high quality cells were available for analysis. Similar to our previous blood and tissue sample findings (15), NK cells were primarily distinguished by NCR3 and KLRK1 with concurrent lack of CD3 expression. Notably, NK cells comprised 15% of the total CD45+ cells, which was greater than the proportion of NK cells in all our previously investigated tissues except for liver (**Figure 1C**). This was also a notably larger percentage of infiltrating tumor NK cells than those seen in canine osteosarcoma (22), which may point to differences between the TME of different cancers, especially osteosarcoma and soft tissue sarcoma with implications for response to therapy (17, 23–27). It’s also necessary to consider that the NK infiltration within the tumor may be a unique feature of this particular sample. We then subset the NK cells in the tumor and integrated them with NK cells from other canine tissues and blood (**Figure 1D**). This new integrated dataset was utilized to conduct further comparisons of tumor NK cells to those in different tissue compartments. The top genes significantly upregulated in tumor NK cells compared to NK cells in all other tissues included genes with a variety of functions, including PLXNA4, known to be upregulated in NK cells that have been reprogrammed after exposure to malignant cells (28) (**Figure 1E**, **Supplementary Table 1**). Tumor NK cells downregulated canonical NK activation markers, such as FASLG, TNFSF10, KLRK1, GZMB, and KLRD1, the latter having both inhibitory and activator capabilities but being a marker of favorable prognosis in human cancers if present in either the serum or tumor (29, 30) (**Figure 1F**). Conversely, canonical NK inhibitory markers were significantly upregulated in canine intra-tumoral NK cells, aligning with previous reports of the inverse relationship between inhibitory genes CD96 and KLRB1 and cytotoxicity signatures in NK cells in human tumors (31).

The genes upregulated in canine STS NK cells versus all other NK cells were used to determine a canine STS NK signature (**Figure 1G**). The most highly expressed genes were associated with chronic activation and cancer pathways, but we also observed significant upregulation of genes associated with adhesion and migration, differentiation and proliferation, and signaling (**Figure 1G**). This pattern was further confirmed when tumor NK cells were compared to canine NK subsets. The six subsets were previously derived from a large population of circulating and tissue-resident NK cells that were categorized based on distinguishing gene expression patterns (15). The d1 cluster was associated with cytotoxicity (ex. GZMB, GZMA, NCR3), the d2 cluster with signaling (ex. SLIT3, KLRF1, SYK), the d3 cluster with regulation (ex. SYNGR1, PDAP1, EID1), the d4 cluster with differentiation (ex. FOXO1, NOTCH2, LEF1), the d5 cluster with proliferation and trafficking (ex. IL2RA, IL7R, ZNF683), and the d6 cluster with inflammation and migration (ex. CTSZ, IFI30, CXCL8).Tumor NK cells were enriched for the d2 and d3 dog NK subclusters, classified by signaling and regulation, respectively (**Figure 1H**). Notably, there was clear overlap in expression characteristics between dog STS and liver NK cells, suggesting potential mixed immunoregulatory and cytotoxic programs in canine sarcoma NK cells consistent with our prior mixed liver NK scRNAseq signature (15). Additionally, a correlation matrix across NK compartments based on previously identified tissue-resident NK signatures, revealed that the only positive correlation present was between liver and sarcoma NK cells (**Figure 1I**) (15). These results appear to corroborate the exhausted phenotype of intra-tumor NK cells seen in human cancers (32). Though tumor-resident NK cells had unique gene signatures in the dog, certain overlapping characteristics with tissue-resident NK cells imply the malleability of NK cell states rather than strict or static functional groups.

### Canine and human NK cells show homology across blood, tissue, and tumor compartments

**Figure 2:**
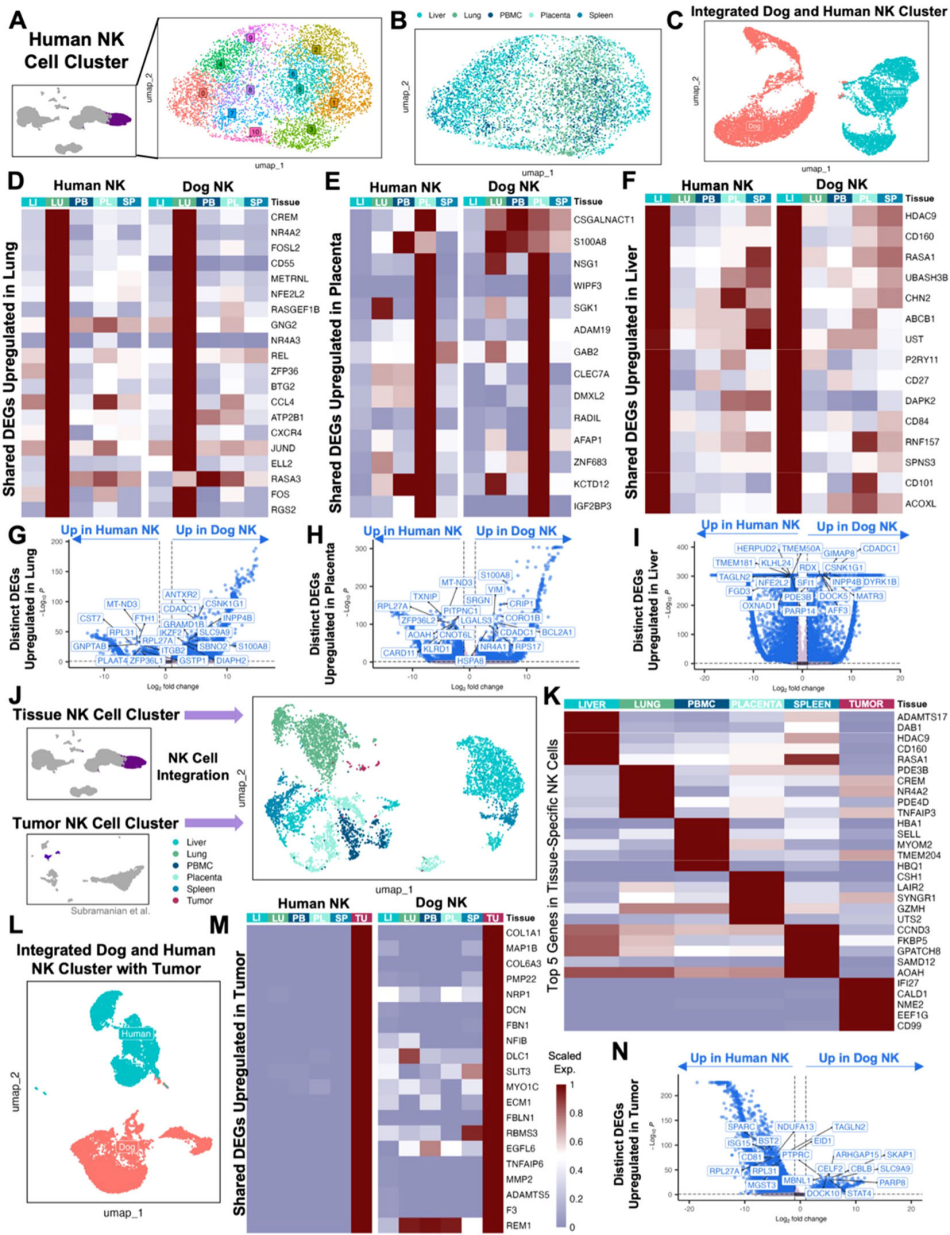
Canine and human NK cells show homology across blood, tissue, and tumor compartments. **(A)** UMAP representation of NK cells subset from the integrated liver, lung, placenta, and spleen dataset with 11 clusters identified by unsupervised clustering. **(B)** UMAP visualization of NK cells color coded by tissue with clustering parameters: res = 1, dims = 1:17. **(C)** NK cells from liver, lung, PBMC, placenta, and spleen tissue in both humans and dogs were integrated and visualized by UMAP, color coded by species. **(D-F)** Genes that were significantly upregulated in NK cells within each tissue compared to NK cells in the remaining tissues within each species with adjusted p-value<0.05 and average log2FC>1 were identified. The average expression of representative genes that were upregulated in both human and dog **(D)** lung, **(E)** placenta, and **(F)** liver NK cells were visualized by heatmap. **(G-I)** Volcano plots with labels for the top ten significant genes sorted by adjusted p value that had expression in at least 10% of cells in both tissues being compared and a log2FC>1 that differentiate human and canine NK cells in the **(G)** lung, **(H)** placenta, and **(I)** liver based on direct DEG comparisons. **(J)** NK cells subset from the integrated human tissue dataset and NK cells subset from a publicly available human UPS sample were integrated and visualized by UMAP color coded by tissue with clustering parameters: res = 1, dims = 1:20. **(K)** Heatmap showing the average expression of the top differentially expressed genes in NK cells from each tissue that distinguish them from NK cells in all other tissues. **(L)** NK cells from liver, lung, PBMC, placenta, spleen, and tumor tissue in both humans and dogs were integrated and visualized by UMAP, color coded by species. **(M)** Genes that were significantly upregulated in tumor NK cells compared to the NK cells in the remaining tissues within each species with adjusted p-value<0.05, average log2FC>1, and at least 10% expression in both tissues were identified. The average expression of genes that were upregulated in both human and dog tumor NK cells were visualized by heatmap. **(N)** Volcano plot with labels for the top ten significant genes that differentiate human and canine NK cells in tumor based on direct DEG comparisons.

Extensive comparative analysis is integral to the continued validation and optimization of the canine model. We therefore sought to further analyze previously published dog and human NK cell datasets for additional comparison. To do so, we subset the identified NK cell clusters from the integrated human blood and tissue dataset and performed unsupervised clustering which resulted in 11 clusters comprising NK cells across tissue compartments, highlighting the complexity of NK cell heterogeneity (**Figures 2A, B**). To more directly compare human and canine NK cells, we integrated NK cells from the liver, lung, PBMC, placenta, and spleen which clustered distinctly based on species (**Figure 2C**). To determine the similarities in tissue-specific genes in NK cells across species, we then analyzed the genes that were significantly upregulated in each tissue in dog and human separately and then identified the overlapping DEGs. There were 98 upregulated genes that overlapped across species in lung NK cells, 18 upregulated genes in placenta NK cells and 15 upregulated genes in liver NK cells (**Figures 2D–F**). Notable genes included CCL4 and CXCR4, which were part of the dog lung NK activation signature and similarly upregulated in human lung, which may point to the distinct role of immune cells, including NK cells, in the lung to coordinate multi-cellular immune responses (15). Likewise, ZNF683 and IGF2BP3 were part of the dog placenta NK differentiation signature and upregulated in human placenta. The activating NK receptor CD160, associated with canine liver NK cells, was similarly upregulated in human liver NK cells. Given these similarities, we then directly compared tissue-resident NK cells across species to understand and highlight important differences (**Figures 2G–I**). Notably, dog placenta NK cells had increased expression of activation and signaling markers, BCL2A1 and CRIP1, compared to human placenta NK cells. However, NK cells in the human placenta upregulated KLRD1 in comparison to dog placenta, a canonical NK marker which functions in NKG2 heterodimers to create either activating or inhibitory signals (33, 34).

Ultimately, the maturation and activation states of NK cells in the peripheral blood and tissue are most relevant to translational analyses of how these cells adapt to or exist in the TME. We therefore accessed a publicly available sample of a human undifferentiated pleomorphic sarcoma (UPS) (20), which served as a human analog of our canine STS sample characterized by histopathology as a spindle cell sarcoma. A single sample was used to mirror the canine STS sample and was chosen based on similarities between canine sarcomas and human undifferentiated sarcoma. The UPS sample of Subramanian et al. included 1,658 CD45+ cells based on a threshold of PTPRC>0. Only 4% of those cells were identified as NK cells, which we then integrated with our human tissue and blood NK cell dataset (**Figure 2J**). The top significant genes in human UPS NK cells were relatively distinct compared to the overlap seen in top genes across other tissues and blood (**Figure 2K**). We next took our total tumor, tissue, and peripheral NK cells from human patients and integrated them with our equivalent canine dataset (**Figure 2L**). There were 172 genes shared between NK cells in canine and human undifferentiated pleomorphic sarcoma that were significantly upregulated compared to their respective canine and human NK cells in other tissue samples (**Figure 2M**). We saw cross-species upregulation of immunoregulatory receptor, NRP1, often expressed in Treg cells but also in NK cells, with potential as a checkpoint target (35, 36). Additionally, COL1A1 and FBLN1 were previously used as markers for canine tumor or fibroblast cells (22), and MMP2 and DCN as markers of mesenchymal-like cells in human cancers (37, 38), with the latter also being a top differentially expressed gene in our canine STS sample (**Figure 1E**). Given that canine and human STS tumor samples had the largest overlap in shared upregulated genes against their respective tissue and blood samples, we used a direct contrast of the tumors to understand the biological relevance of top DEGs (**Figure 2N**). Human UPS NK cells had increased expression of EID1, one of the markers of the regulatory canine NK subcluster d3, and ISG15, part of the differentiation signature in canine placenta NK cells (15). Also, human sarcoma-infiltrating NK cells expressed BST2, which has been observed to be upregulated in blood cancer cell lines exposed to NK cells (39). On the other hand, canine tumor NK cells had increased expression of PTPRC/CD45, a hallmark present on all leukocytes, which had a high probability of interaction with MRC1 on myeloid cells in the canine lung (15). STAT4, also significantly increased, is an epigenetic regulator for NK cells involved in NK activation and IFN-γ production (40). Together, we observed subtle differences between NK cells in the TME across species that point to modifications in activation states with potential influence of neighboring cells. Nevertheless, the considerable similarities in NK cell DEG between sarcoma samples between dog and human support the validity of the canine model in STS research, aligning with previous reports of conserved characteristics across canine and human cancers (5, 8, 9, 12, 41–43).

### NK proportions increase in response to treatment in good responders to first-in-dog immunotherapy regimens

**Figure 3:**
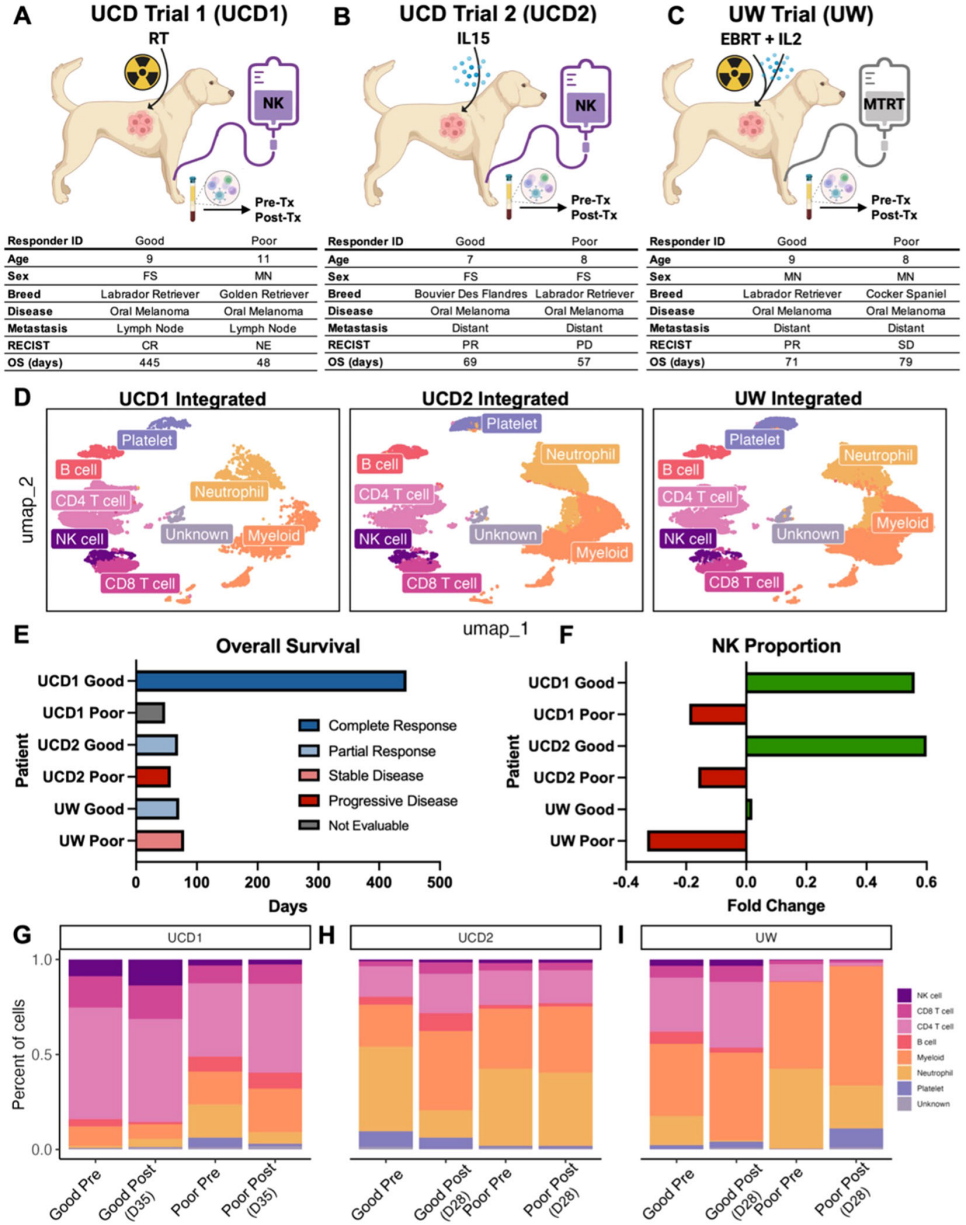
NK proportions increase in response to treatment in good responders to first-in-dog immunotherapy regimens. **(A–C)** Schema depicting clinical trial treatments and corresponding characteristics and outcomes for dogs enrolled in **(A)** UCD trial 1 with combination of radiotherapy and allogeneic NK cell transfer, **(B)** UCD trial 2 with combination of inhaled IL-15 and autologous NK cell transfer, and **(C)** UW trial with combination molecular targeted radionuclide therapy (MTRT), IL-2 cytokine, and external beam radiation therapy (EBRT). Good and poor responders were determined based on RECIST criteria of the primary tumor and overall survival. PBMCs were collected pre and post treatment for each dog. The UCD1 Good responder did not survive long enough to determine response and was therefore considered not evaluable (NE). *UW good responder was lost to follow up at 71 days. **(D)** UMAP visualizations of the twelve integrated PBMC samples split by trial and color coded by cell type. **(E)** Bar plot depicting the overall survival or date or last follow up (UW Good) for each of the dogs included in the analysis. Bars are color coded by response based on RECIST criteria. **(F)** Bar plot showing fold change of the NK cell proportion in each dog included in the analysis. Green and red bars represent positive and negative fold change respectively. Fold change of NK cell proportion was calculated by (Post-treatment – Pre-treatment)/Pre-treatment. **(G–I)** Bar plots showing the percent of each cell type in each PBMC sample included in the analysis. Plots are split by trial and then further split by pre and post treatment sample for each dog. Post treatment samples were obtained **(G)** 35 days after the start of treatment in UCD trial 1, **(H)** 28 days after the start of treatment in UCD Trial 2, and **(I)** 28 days after the start of treatment in UW trial.

Insights into blood, tissue, and tumor-infiltrating NK cells are most relevant in the larger scope of clinical data, including how to incorporate these results to inform future clinical trials. We therefore analyzed blood samples from dogs who were enrolled on three separate, NK-targeting trials. Dogs in UCD Trial #1 (UCD1) underwent four weekly treatments of palliative radiotherapy (RT) in addition to an infusion of PBMC-derived allogeneic NK cells immediately following the fourth and final RT for buccal melanoma (16) (**Figure 3A**). Dogs in UCD Trial #2 (UCD2) received two infusions of autologous NK cells 7 days apart in combination with inhaled rhIL-15 simultaneously for a total of 14 days for dogs with gross pulmonary metastases from osteosarcoma and melanoma (16) (**Figure 3B**). These two trials were the first to employ systemic administration of PBMC-derived NK cells in dogs with naturally occurring cancer (16). We also analyzed samples from dogs in a third trial, the University of Wisconsin (UW) cohort, where dogs with melanoma were treated with low-dose molecular targeted radionuclide therapy (MTRT), external beam radiation therapy (EBRT) and intratumoral injection of GD2/IL-2 fusion immunocytokine (**Figure 3C**). These dogs were treated based on the results of a protocol cohort that demonstrated safety and feasibility of the combination therapy (44). Together, PBMCs were available for six dogs, two from each trial representing a good responder and a poor responder. For each dog, a pre-treatment and post-treatment PBMC sample was obtained for a total of 12 PBMC samples which were submitted for scRNAseq. The pre-treatment sample was obtained prior to any treatment, including radiation therapy, for a true baseline. The post-treatment sample was obtained 28–35 days following the initiation treatment in all trials. Therefore, the post-treatment sample in the UCD1 cohort was four weeks following first RT and two weeks following NK transfer and in the UCD2 cohort was four weeks following the first NK transfer and initiation of inhaled IL-15. Those samples were integrated, with an average of 5,425 cells per sample, and cell types identified manually by canonical cell markers (**Figure 3D**). Samples were then assessed and compared based on trial, treatment timepoint, and response, which was assessed based on RECIST criteria and overall survival (**Figure 3E**). In all clinical trial dogs analyzed, death was disease related. The poor responder from UCD1 did not live long enough to determine response and therefore was not evaluable by RECIST criteria but died from tumor progression after 45 days. The good responder from UCD1 had a complete response and the longest survival of 445 days. In the UW trial, the good responder had a shorter overall survival than the poor responder. This points to the differences between response and overall survival, especially in veterinary medicine when humane euthanasia may be elected by the medical team or by dog owner for a variety of reasons. Responses corresponded reliably with the fold change of NK cell proportion following treatment, with all three poor responders showing a negative fold change in NK cells post therapy and all three good responders showing a positive fold change (**Figure 3F**). The proportion of peripheral blood NK cells was highest in UCD1, reaching a maximum of 13.7% of cells post treatment (**Figure 3G**). NK cell frequencies ranged between 1-2% of total cells in UCD2 and comprised fewer than 1% of total cells in the UW poor responder (**Figures 3H, I**). Overall, we observed variability of NK proportions in the peripheral blood of dogs diagnosed with cancer, but evidence indicating that dogs with a positive fold change in NK cell frequencies post treatment correlates with favorable response.

### Good responders upregulate NK activation signatures after treatment while poor responders have minimal treatment-related changes

**Figure 4:**
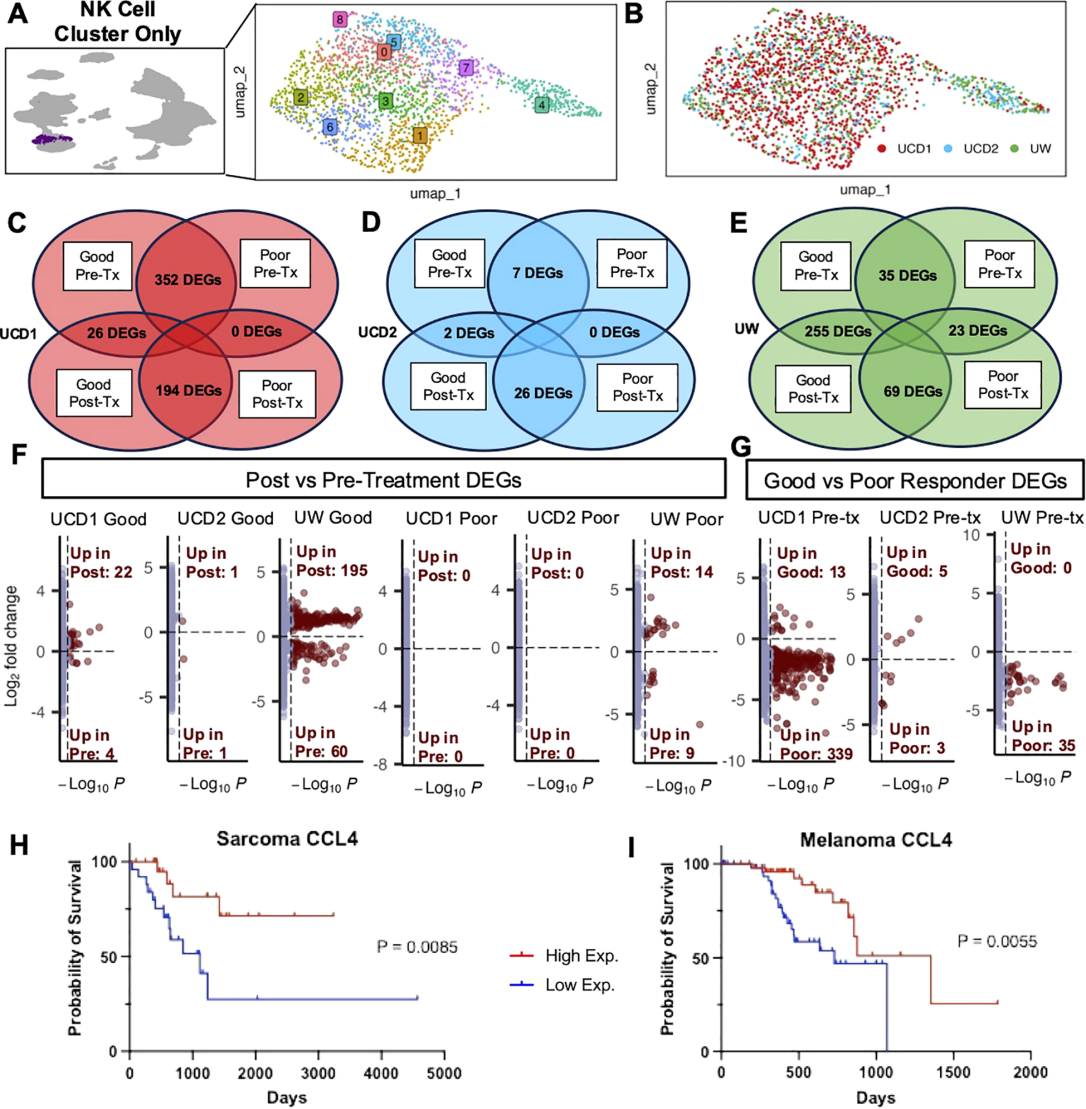
Good responders upregulate NK activation signatures after treatment while poor responders have minimal treatment-related changes. **(A)** UMAP representation of NK cells subset from the integrated PBMC samples across trials with 9 clusters identified by unsupervised clustering. **(B)** UMAP visualization of NK cells color coded by trial. **(C–E)** DEG analysis was completed between post and pretreatment for each dog and between good and poor responders for each timepoint. The number of genes with adjusted p-value<0.05 in each comparison are depicted in Venn diagrams for **(C)** UCD trial 1, **(D)** UCD trial 2, and **(E)** UW trial. **(F, G)** Volcano plots distinguish the number of genes that were significantly upregulated or downregulated in **(F)** post vs pre-treatment and **(G)** good vs poor responders pre-treatment. **(H, I)** Kaplan–Meier survival curve showing the survival time of TCGA UPS **(H)** and TCGA Melanoma **(I)** patient samples with high and low expression of CCL4, which was significantly upregulated both UCD1 and UW good responders in response to treatment in addition to the activated lung signature.

Given our intriguing results implicating the role of NK cells in treatment response, we then specifically analyzed the NK cell cluster from these patients to focus on their distinct NK characteristics (**Figure 4A**). NK cells from these patients included over 2,000 cells across trials that met quality standards for analysis (**Figure 4B**). We completed DEG analysis comparing pre- and post-treatment samples in good responders and poor responders to hone in on differences due to the treatment itself and compared good vs poor responders at pre- and -treatment timepoints in all trials to explore differences between the dogs with potential biomarkers of response. (**Figures 4C–E**). We found that there were large differences in pre-treatment samples between poor and good responders in UCD1 (palliative RT plus allogeneic PBMC-derived NK cells) and that the poor responder had no genes upregulated in response to treatment (**Figure 4C**). Subjects from UCD2 (autologous PBMC-derived NK cells plus inhaled IL-15) showed minimal changes overall, both in response to treatment and between responders, suggesting limited immune effects systemically from this immunotherapy regiment (**Figure 4D**). This contrasted with the UW trial (MTRT with immunocytokine) which demonstrated 255 differentially expressed genes post treatment in the good responder and, similar to UCD1, had considerably fewer DEG changes (23) in the poor responder (**Figure 4E**). Overall, we observed that the majority of DEGs between pre and post treatment in good responders were significantly upregulated in response to treatment rather than downregulated (**Figure 4F**). Additionally, the number of genes that were different between good and poor responders at the pre-treatment time point varied by trial, suggesting that upregulation of specific gene signatures at baseline contributes to the immune changes among favorable and unfavorable responders with potential downstream effects on clinical outcomes (**Figure 4G**).

Given these results, we sought to explore the specific genes contributing to these differences in more detail. We evaluated key genes that were upregulated post treatment in good responders and key genes upregulated at baseline in poor responders, with specific emphasis on genes that were previously identified in our tissue-specific signatures (15) (**Supplementary Figure 1**). Most striking was the identification of CCL4, which was significantly upregulated in good responders from both UCD1 and UW and was also identified in activated lung NK cells in both dog and human scRNAseq analysis (**Figure 2D**). Additional genes upregulated in good responders included CX3CR1 and ITGAL in the UCD1 trial and GZMB and KLRK1 in the UW trial (**Supplementary Figure 1**). These data coincided with data from The Cancer Genome Atlas (TCGA), where greater CCL4 expression in Melanoma and UPS human tumor samples was significantly associated with improved survival (**Figures 4H, I**). Although poor responders had minimal response to treatment overall, we did see that they expressed several activation genes at greater levels than good responders before the start of treatment, including BCL2A1, present in both UCD1 and UW trials. The overall pattern of our results points to the possibility that response to treatment may be associated with changes in NK cell gene signatures during cancer immunotherapy, although this may vary by cancer type and immunotherapy regimen.

## Discussion

Here we report a canine sarcoma-infiltrating NK cell signature in combination with a direct comparison to human and canine NK gene expression across tissues known to harbor tissue resident NK cells, including blood, spleen, liver, lung, placenta. Canine sarcoma-infiltrating NK cells exhibited a dysregulation-associated signature with the most similarity to tissue-resident NK cells from the liver. Importantly, there was striking cross-species similarities in tissue resident NK cells between dogs and humans. For both species, we observed heterogeneous gene signatures in tumor-infiltrating NK cells with features of both activation and exhaustion, suggesting a lack of reproducible and adaptive DEG by NK cells in the sarcoma TME. Finally, we reveal changes in peripheral NK cell gene expression that align with response to NK-targeting approaches in three first-in-dog immunotherapy trials. Notably, upregulation of CCL4 was a consistent finding in activated NK cells, seen in human and canine lung NK signatures as well as in response to treatment in dogs that responded well to immunotherapy (15). Together, we describe transcriptomic responses to first-in-dog NK-targeting clinical trials in the context of tissue and tumor NK signatures with translational relevance to immune-oncology research for both dogs and people.

The complexity of the TME continues to be a prominent topic in immune-oncology research, the unraveling of which is necessary for the improvement of therapeutics available for solid cancers. Immune cells within the TME undergo maladaptive changes, shifting to dampened or pro-tumor responses. This is especially apparent in our own demonstration of the mixed activated and exhausted phenotype of NK cells in canine soft tissue sarcoma, representing the maladaptive signals present in the TME. Tumor NK cells corresponded most with liver NK cells, which expressed similar inhibitory markers, such as CD96. The population of inhibitory NK cells in the liver could potentially be part of typical and necessary regulation of immune responses in normal tissue that are exploited during tumor progression. Overall, the various states or subsets of NK cells point to inherent malleability which may underlie the difficulties in applying NK cell therapies clinically given apparent susceptibility to local cues impacting their phenotype and function but also may point the way to harness them for improved anti-cancer efficacy.

Integrated analysis of NK cells from dogs enrolled in canine NK-targeting clinical trials showed distinct signatures with greater DEGs related to activation and recruitment post-treatment in responders compared to non-responders. Good responders also generally had fewer activation markers at baseline than poor responders but had significantly greater responses to treatment. Numerous studies have shown that baseline gene expression can predict responses to immunotherapy in several human cancers (45–47). However, others have shown that predictive biomarkers of response are more distinct during, or post-treatment, compared to pre-treatment samples (48, 49). A potential explanation of this pattern is that NK cells with increased plasticity are more capable of adjusting their activity and effector functions in response to treatment, leading to better anti-tumor responses. The tunability of NK cells corresponds well with our data regarding tissue and tumor-specific NK cells. Importantly, the malleability of NK subsets seems to be a conserved characteristic between canine and human NK cells.

It is important to note these data presented in this paper are not exhaustive and there are inherent limitations that should be acknowledged when interpreting the results. While we were able to obtain multiple canine and human tissue types, there were only two samples for each canine organ and only a single sample for human organs and tumor. Therefore, it is necessary to note the potential impact of donor variability and the need for future studies to confirm these findings with additional samples and additional tumor types. Furthermore, all clinical trial dogs were from NK-targeting immunotherapy trials, but the mechanism of anti-tumor effect elicited by NK cells varied notably. The differences between the trials may be impacted by implicit distinctions between adoptive cell therapy versus radiotherapy and the potential for differential immune effects when using IL-2 versus IL-15. Also, we only analyzed two dogs from each trial potentially impacting the representativeness of our data. Though there are clear limitations to this study, the analyses presented here add important data improving the characterization of canine NK cells, especially tissue and tumor resident NK cells and their comparative and translational context.

Overall, we shed light on the diverse heterogeneity of canine NK cells across tissues with features of adaptability that appear to be both adaptive to tissue residence and maladaptive to the TME. Our unique analysis of NK cell samples from dogs enrolled on canine immunotherapy trials elucidates a potential survival trend correlated with post-treatment increases in NK cell abundance and serves as a blueprint of how NK subset identification and characterization can increase our understanding of gene expression changes. Ultimately, our study serves as a basis for advancing mechanistic investigations into novel NK cell therapeutic approaches for both dogs and people.

## Data Availability

The data presented in the study are deposited in the National Center for Biotechnology Information Sequence Read Archive repository, accession number PRJNA1259073.
