# Supplementary figures and images for: Comparative transcriptomic analysis of tumor- infiltrating canine natural killer cells and candidate biomarkers from first-in-dog NK immunotherapy trials
Source: Front Immunol. 2025 Oct 24;16:1646849. doi: 10.3389/fimmu.2025.1646849 (PMC12592164; doi:10.3389/fimmu.2025.1646849)

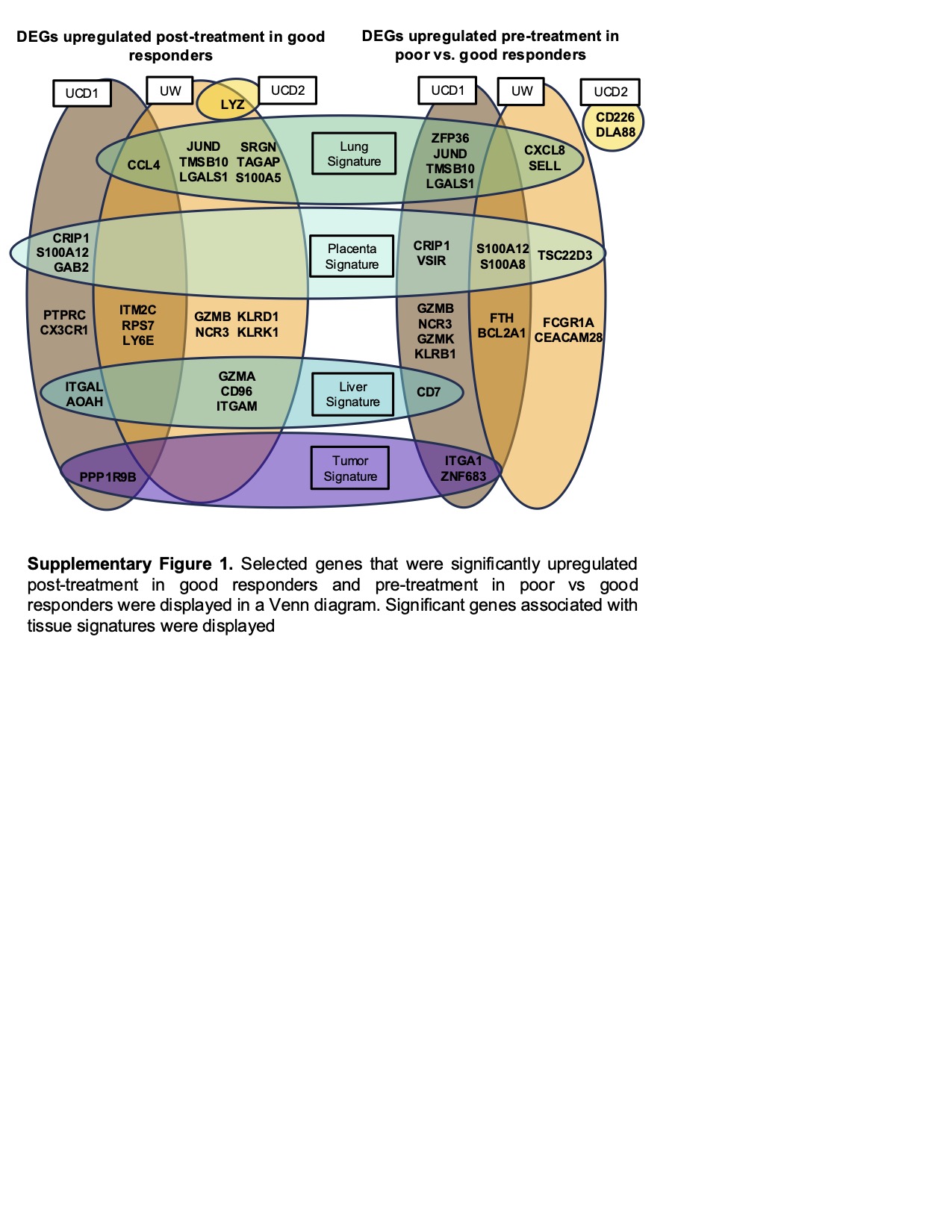

Supplement: Supplementary file 1 [file Image1.jpeg]
